# Antecedents of Interest and the Investment of Fluid Intelligence in the Formation of Crystalized Intelligence

**DOI:** 10.3389/fpsyg.2021.679504

**Published:** 2021-10-04

**Authors:** Yoav Ganzach

**Affiliations:** ^1^Ariel University, Ariel, Israel; ^2^The Department of Economic and Business Administration, Tel Aviv University, Tel Aviv, Israel

**Keywords:** fluid intelligence, crystalized intelligence, investment traits, intelligence development, interaction

## Abstract

Most of the studies of the effects of fluid intelligence and non-cognitive characteristics on crystalized intelligence examined additive effects. The results of the few studies that examined interactive effects are inconsistent. Some find a positive (facilitating) interaction and some find a negative (compensatory) interaction. We improve on these previous studies by examining non-cognitive characteristics that were not studied before and by using a very large representative sample (*n* = 11,266). We find a positive/facilitating interaction. We discuss the implication of these results to theories about the joint effect of fluid intelligence and non-cognitive characteristics on crystalized intelligence.

## On the Interactive Effects of Investment Traits and Fluid Intelligence in the Formation of Crystalized Intelligence

Cattell (1943) distinguished between fluid intelligence that represents a “purely general ability to discriminate and perceive relations between any fundaments, new or old” and crystallized intelligence that “consists of discriminatory habits long established in a particular field, originally through the operation of fluid ability, but no longer requiring insightful perception for their successful operation.” (p. 178). Fluid intelligence is thought to be related to fundamental cognitive processes such as speed of processing and inspection time, while crystalized intelligence is thought to be related to information acquired in diverse areas such as mathematical knowledge, verbal knowledge and mechanical knowledge (Schmidt, 2011). According to Cattell, crystalized intelligence is determined by fluid intelligence and by non-cognitive individual differences that influence the acquisition of knowledge: “this year's crystallized ability level is a function of last year's fluid ability level – and last year's interest in school work and abstract problems generally” (p. 139). In latter research these individual differences were conceptualized as investment traits–personality characteristics and specific interests that determine the extent to which people invest their time and effort in their intellect (Ackerman, 1996).

Although the original formulation of Cattell's theory had a clear interactive aspect to it, in that the extent to which fluid intelligence is invested in the formation of crystalized intelligence was assumed to depend on the levels of the investment traits, most of the models of the joint effects of fluid intelligence and investment traits on crystalized intelligence have been additive. For example, the PPIK (Process, Personality, Interests, and Knowledge, Ackerman, 1996) the most popular model about these effects, proposes only additive relationships between these variables. Furthermore, in the most extensive review of the literature on investment traits, Von Stumm and Ackerman (2013) did not report any study of non-additive relationship, and in particular, any study of interactive relationships (see for example Table 3 p. 853–855). This approach also characterizes more recent research (e.g., Thomas et al., 2016; Luong et al., 2017; Powell et al., 2017; Von Stumm, 2018).

### The Facilitating Effect of Between Fluid Intelligence and Investment Traits

**Figure 1:**
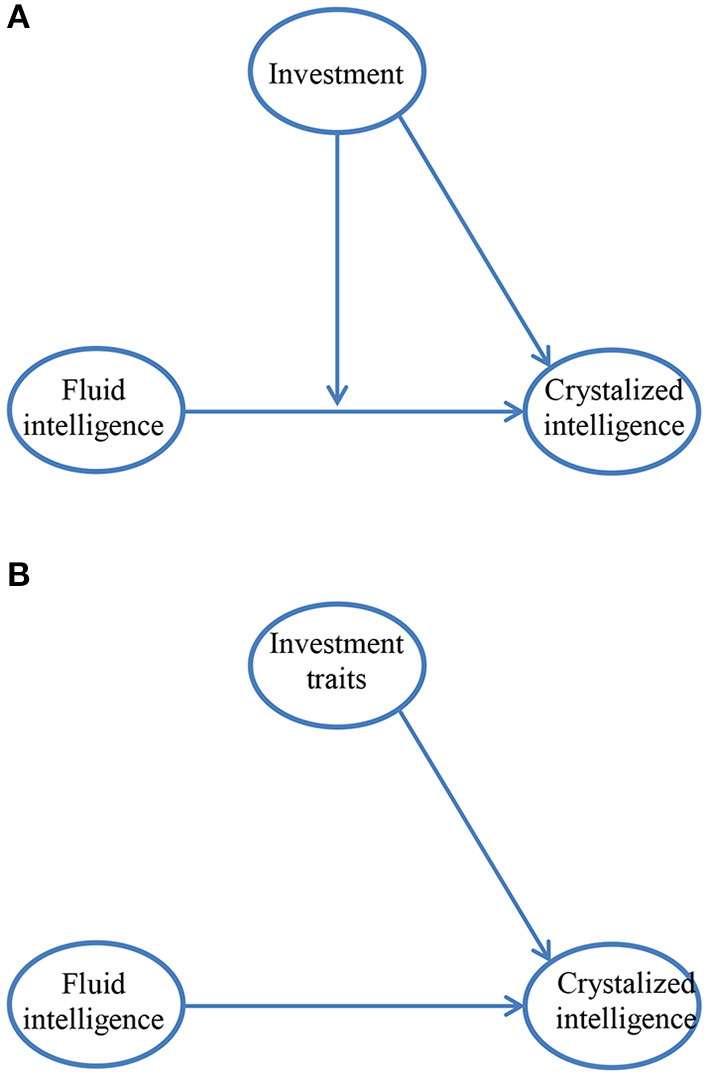
**(A)** An interaction model of the relationship between fluid and crystalized intelligence. **(B)** An additive model of the relationship between fluid and crystalized intelligence.

Within Cattell's theory, the interactive model of the effect of fluid intelligence and investment on crystalized intelligence is a moderation model. It suggests that the effect of fluid intelligence is stronger when investment is high than when it is low, or alternatively that the effect of investment on crystalized intelligence is moderated by fluid intelligence, it is stronger when fluid intelligence is high than when it is low. Figure 1A presents the framework by which investment is conceptualized in an interactive model as opposed to the framework by which it is conceptualized in the additive model (Figure 1B).

Another way to view the interaction between investment and fluid intelligence is that it is associated with a facilitating (synergistic) effects that investment has on fluid intelligence in the acquisition of knowledge; that is, with the idea that, with regard to the development of crystalized intelligence, fluid intelligence may be futile if it is not invested in the acquisition of knowledge. Alternatively, this interaction may be viewed as the result of a facilitation effect of fluid intelligence leading to a more efficient channeling of investment resources; that is with the idea that investment may be futile if fluid intelligence is missing. In this respect, the effects of intelligence and investment on the acquisition of knowledge is similar to the effects of ability and motivation on performance, effects which are associated with the idea that people will not act if their actions do not serve relevant goals (Locke and Latham, 1990) and that differences in ability are observed only when motivation to perform is high (Duckworth et al., 2011); or more generally, with the notion that the effect of ability on performance is moderated by motivation (or alternatively that the effect of motivation on performance is moderated by ability), the cornerstone of expectancy theories of motivation (Vroom, 1964; Peterson et al., 1993).

### Literature Review

Table 1 presents a summary of a literature review of the few studies that examined the interaction between investment traits and various measures that were considered by the researchers to be proxies of fluid intelligence. It is evident from the table that there are considerable inconsistencies in the results. About half of the studies yielded positive interactions and about half yielded non-significant or negative interactions. Note that all the negative interactions were obtained in studies that were conducted within the framework of the OFCI model (the Openness-Fluid-Crystallized-Intelligence model. Ziegler et al., 2012, 2015) which view openness to experience as a central investment trait and suggest that its interaction with fluid intelligence affects crystalized intelligence. However, in contrast to the spirit of Cattell's theory, which implies a facilitating (positive) interaction, the OFCI model proposes that this interaction is compensatory (negative)^1^.

**Table 1:** Summary of studies examining interactions between investment traits and intelligence tests on crystalized intelligence.

**Paper**	***n***	**Investment trait**	**Measure of fluid intelligence**	**Measure of crystalized intelligence**	**Design**	**Result^a^**
Ziegler et al. (2009)	271	Conscientiousness	Basic module from the Intelligence-Structure-Test-2000-R	GPA	Between	+
Ziegler et al. (2009)		Achievement striving	Basic module from the Intelligence-Structure-Test-2000-R	GPA	Between	0
Ziegler et al. (2012) Study 1	180	Openness	Basic module of the Intelligence Structure Test 2000 R	Lexical Knowledge Test	Between	**_**
Ziegler et al. (2012) Study 2	172	Openness	CFT-2	The Wechsler's vocabulary test	Within	0
Heaven and Ciarrochi (2012)	786	Openness	Verbal and numerical ability tests	GPA	Between	+
Di Domenico and Fournier (2015)	271	Conscientiousness	Wonderlic test	GPA	Between	+
Di Domenico and Fournier (2015)	271	Autonomous motivation	Wonderlic test	GPA	Between	0
Zhang and Ziegler (2015)	836	Openness	Raven progressive matrices	GPA	Between	**_**
Zhang and Ziegler (2015)	836	Conscientiousness	Raven progressive matrices	GPA	Between	0
Bergold and Steinmayr (2018)	664	Conscientiousness	Basic module from the Intelligence-Structure-Test-2000-R	GPA	Between	+
Strobel et al. (2019)	290	Need for cognition	Basic module from the Intelligence-Structure-Test-2000-R	GPA	Between	_
Lechner et al. (2019)	4,626	Interest	NEPS matrices test	NEPS reading and math	Within	+

*For the CGT-2 see Cattell and Weiss (1980), for the Intelligence-Structure-Test 2000 R see Liepmann et al. (2007), for the verbal and numerical ability tests see Heaven and Ciarrochi (2012), for the NEPS tests see https://www.neps-data.de/Mainpage, for the Wonderlic test see Wonderlic Inc (2002)*.

a *+: significant positive interaction, −: significant negative interaction, 0: non-significant interaction*.

There are, however, a number of issues with the studies that are reviewed in this Table. First, some of the studies used general intelligence tests rather than tests of fluid intelligence as independent variable. Second, most of them used school grades rather than tests of crystalized intelligence, as dependent variable (see Borghans et al., 2011; Lechner et al., 2017, for the distinction between the two). Second, except of one study (Lechner et al., 2019) none of the studies in Table 1 included quadratic effects of the components of the interaction as controls. It is well-known, however, that when quadratic effects are not included, estimated interaction may be biased (Lubinski and Humphreys, 1990; MacCallum and Mar, 1995) and even lead to estimated coefficients which are opposite in sign to the true coefficients (Cortina, 1993; Ganzach, 1997), which may explain the negative interactions that were obtained in previous research (Table 1). Finally, and most importantly, because the power to detect interaction effects is low (Aguinis and Stone-Romero, 1997; Shieh, 2009), the sample sizes of most of these studies may have been too small to reliably detect our focal interaction effects.

In view to these issues, a central purpose of the current study is to estimate interactive effects involving the relationship between fluid intelligence and crystalized intelligence based on a large database using appropriate measures of fluid and crystalized intelligence and controlling for the quadratic effects of the interaction's components.

### Antecedents of Interest

Whereas, the idea that the investment of fluid intelligence is involved in the formation of crystalized intelligence was examined primarily with regard to investment traits, it is possible that other individual characteristics are also involved in an interactive investment process. Within the intelligence literature we could find only one example in which individual characteristics other than investment traits were suggested as moderators of the effect of fluid intelligence on crystalized intelligence. Reasoning that high SES individuals are more motivated to invest their time and attention in learning experiences that are consistent with their interests, and using a large database that included information about 375,000 students, Tucker-Drob and Briley (2012) found that socioeconomic status moderates the interest-knowledge associations such that this association is stronger for individuals living in higher rather than lower socioeconomic areas.

Viewing socioeconomic status as a proxy for interest, Tucker-Drob and Briley's (2012) study suggests that demographic proxies of interest may be helpful in examining the investment aspects of crystalized intelligence. Furthermore, Tucker-Drob and Briley's (2012) study also suggest that these demographic proxies may be particularly important in studying the interactive features of investment. The reason for this is that the low power of detecting interactions requires reliance on large databases which usually include demographic information, but not necessarily information about investment traits. Indeed, it is no wonder that Tucker-Drob and Briley (2012) study was based on a large database of 375,000, and that the other study that provide the most reliable evidence for the interactive features of investment (among other things by controlling for quadratic effects and using a repeated measure design) – Lechner et al. (2019) – was also based on a large sample of 4,626 participants)^2^^,^^3^.

Thus, our focus in the current paper is how the effect of fluid intelligence on crystalized intelligence is moderated by two demographic variables: Parents' education and sex. In addition, viewing educational aspiration as directly related to socio-economic status, and because its availability in our data, we also examine the moderation effect of this variable. All these three variables could be viewed as antecedent of interest, and therefore as proxies of interest in estimation its moderation of the relationship between fluid and crystalized intelligence. Parents education is a moderator because it is associated with the conditions that facilitate/hinder knowledge acquisition: Growing-up with highly educated parents provide people with more opportunities to use their crystalized intelligence in acquiring the knowledge underlying the success in crystalized intelligence tests. Educational aspiration reflects the motivation for knowledge acquisition. For example, in a review of the literature about aspirations and expectations of students, Saha (1997) writes that “Although ambition and motivation tend to be general concepts, they are usually operationalised in terms of some future objective or objectives which are seen as desirable by students. Most often these general notions specifically focus on educational and occupational goals which the students, at least at the time, claim to strive toward.” (p. 513). Finally, sex is a likely moderator of the relationship between fluid and crystalized intelligence because of gender differences in interest: males are more interested in technical and mathematical skills and females are more interested in verbal skills (e.g., Su et al., 2009; Stoet and Geary, 2020).

In this respect there is a fundamental difference between our hypotheses regarding the moderation effects of parents' education and educational aspirations and the moderation effect of sex. Whereas, the first two are hypothesized to increase the effect of fluid intelligence on all crystalized abilities, sex may have an opposite moderation effect on different abilities because of the greater interest of males [females] in technical [verbal] skills (Wang et al., 2013): Fluid intelligence may have a stronger effect on verbal abilities among females than among males, and a weaker effect on mathematical and technical abilities among males than about females. That is, if females are coded as 0 and males as 1, we should expect that the Sex × fluid-intelligence interaction will be negative with regard to verbal abilities and a positive with regard to mathematical and technical abilities.

## Method

### Data

The data were taken from the 1979 cohort of the National Longitudinal Survey of Youth (NLSY), conducted with a probability sample of 12,686 Americans (with an oversampling of Afro-Americans, Hispanics and economically disadvantaged whites) born between 1957 and 1964 (11,266 subjects without missing values on the relevant variables). The mean age of the participants when they took the cognitive ability tests in 1980 was 18.66 (std = 2.25). Because of missing values, the number of subjects in the regression analyses was 11,448. The data are available at https://www.nlsinfo.org/content/cohorts/nlsy79.

### Cognitive Ability Measures

Cognitive abilities were measured by the *Armed Services Vocational Aptitude Battery* (ASVAB), which included 10 tests: coding speed, numerical operations, general science, arithmetic reasoning, mathematical knowledge, word knowledge, paragraph comprehension, auto and shop information, mechanical comprehension, and electronics information. ASVAB scores were based on item response theory statistics, with higher scores indicating better performance. To facilitate interpretation, ASVAB scores were standardized prior to the analyses.

Since the measures available in this data set did not allow for deriving a pure measure of fluid intelligence, we relied on the idea that fluid intelligence is linked to processing speed (Fry and Hale, 1996, 2000; Osmon and Jackson, 2002; Zimprich and Martin, 2002; Coyle et al., 2011; but see Roberts et al., 2000), and used the average of the two scores of the speeded tests of the ASVAB–coding speed and numerical operations – as a measure of fluid intelligence. The rest of the tests are associated with knowledge-related abilities in specific areas: math/science ability (the tests in general science, arithmetic reasoning, and mathematical knowledge), verbal ability (word knowledge), and technical ability (auto/shop information (mechanical comprehension, and electronic information), and these were our measures of crystalized abilities.

### Antecedents of Interest

Educational aspirations were measured by subjects' responses to the question “how many years of education they would like to complete?” (mean = 14.39, std = 2.30); parents' education was the mean of the highest grade completed by the father and mother (mean = 10.83, std = 3.27). Sex was coded as 1 for males and 0 for females.

## Results and Discussion

Table 2 presents means and inter-correlations of our variables.

**Table 2:** Inter-correlations of dependent and independent variables.

	***N***	**Mean**	**STD**	**1**	**2**	**3**	**4**	**5**	**6**	**7**	**8**	**9**	**10**	**11**	**12**
1. Fluid intelligence	11,878	0	1	–											
2. General science	11,878	0	1	0.58	–										
3. Arithmetic reasoning	11,878	0	1	0.64	0.73	–									
4. Mathematical knowledge	11,878	0	1	0.62	0.70	0.82	–								
5. Word knowledge	11,878	0	1	0.67	0.82	0.72	0.69	–							
6. paragraph comprehension	11,878	0	1	0.67	0.72	0.70	0.67	0.82	–						
7. Auto/shop information	11,878	0	1	0.38	0.68	0.59	0.47	0.61	0.51	–					
8. Mechanical comprehension	11,878	0	1	0.46	0.71	0.70	0.62	0.64	0.57	0.75	–				
9. Electronic information	11,878	0	1	0.48	0.77	0.68	0.60	0.72	0.63	0.76	0.75	–			
10. Age	11,878	0	1	0.17	0.20	0.17	0.09	0.23	0.19	0.24	0.17	0.25	–		
11. Parents' education	12,686	19.66	2.25	0.36	0.45	0.40	0.43	0.46	0.41	0.30	0.35	0.37	0.03	–	
12. Educational aspiration	12,241	10.83	3.27	0.39	0.41	0.41	0.47	0.44	0.41	0.20	0.28	0.31	0.10	0.36	–
13. Sex	12,624	0.50	0.50	−0.17	13	0.10	0.02	−0.02	−0.10	0.43	0.31	0.29	−0.01	0.02	−0.01

*Coding of sex is 1 for males, 0 for females*.

To examine our interaction hypothesis, we regressed each of the eight crystalized abilities on fluid intelligence, our focal investment trait variables and the interaction between fluid intelligence and each of the three investment traits. In addition, our regressions also include the quadratic terms of the independent variables to account for a possible overlap between interaction and quadratic terms. To facilitate interpretability of the main effects, all independent variables were mean centered.

The results of these eight regressions are presented in Table 3. By and large, the results are consistent with our hypothesis: (1) three of the eight fluid intelligence × parents' education interactions were significantly positive whereas the remaining five interactions were not significantly different from zero; (2) five of the fluid intelligence × educational aspiration interactions were significantly positive whereas the remaining four interactions were not significant; (3) consistent with the idea that males, more than females, invest their fluid intelligence in math/science and in technical abilities, the interactions between fluid intelligence and sex were significantly positive for the three math/science abilities as well as the three technological abilities. For the two verbal abilities, the interactions between fluid intelligence and sex were non-significant^4^.

**Table 3:** Regressions of crystalized abilities.

	**Math/science abilities**						**Verbal abilities**				**Technical abilities**					
	**General science**		**Arithmetic reasoning**		**Math knowledge**		**Word knowledge**		**Paragraph comprehension**		**Auto/shop information**		**Mechanical comprehension**		**Electronic information**	
	**B**	**Sderr**	**B**	**Sderr**	**B**	**Sderr**	**B**	**Sderr**	**B**	**Sderr**	**B**	**Sderr**	**B**	**Sderr**	**B**	**Sderr**
Intercept	0.051^**^	0.013	−0.092^**^	0.013	−0.128^**^	0.013	0.086^**^	0.012	0.093^**^	0.013	0.132^**^	0.014	0.03^**^	0.014	0.057^**^	0.014
Fluid intelligence (Fl)	0.469^**^	0.009	0.630^**^	0.009	0.582^**^	0.009	0.522^**^	0.008	0.553^**^	0.009	0.387^**^	0.009	0.458^**^	0.009	0.435^**^	0.009
Parents' education (PE)	0.073^**^	0.002	0.052^**^	0.002	0.060^**^	0.002	0.068^**^	0.002	0.053^**^	0.002	0.046^**^	0.003	0.058^**^	0.003	0.059^**^	0.003
Education aspiration (EA)	0.060^**^	0.003	0.056^**^	0.003	0.098^**^	0.003	0.061^**^	0.003	0.053^**^	0.003	−0.013^**^	0.003	0.012^*^	0.004	0.024^**^	0.004
Sex	0.404^**^	0.014	0.394^**^	0.013	0.220^**^	0.013	0.133^**^	0.013	−0.016	0.013	0.999^**^	0.014	0.763^**^	0.015	0.718^**^	0.014
Age	0.049^**^	0.003	0.022^**^	0.003	−0.019^**^	0.003	0.060^**^	0.003	0.037^**^	0.003	0.080^**^	0.003	0.040^**^	0.003	0.077^**^	0.003
FI^2^	−0.089^**^	0.008	0.038^**^	0.008	0.050^**^	0.008	−0.123^**^	0.007	−0.106^**^	0.008	−0.082^**^	0.008	−0.042^**^	0.008	−0.060^**^	0.008
PE^2^	0.001^*^	0.000	0.002^**^	0.000	0.003^**^	0.000	0.001^*^	0.000	0.001	0.000	−0.001^*^	0.000	0.001	0.000	0.000	0.000
EA^2^	0.002	0.001	0.003	0.002	0.004^*^	0.001	0.003	0.001	0.002	0.001	−0.005	0.001	−0.002	0.001	0.001	0.001
Age^2^	0.000	0.001	0.001	0.001	−0.001	0.001	0.000	0.001	−0.002	0.001	−0.001	0.002	0.001	0.002	0.000	0.002
**FI** × **PE**	0.005	0.003	0.013^**^	0.003	0.013^**^	0.003	0.003	0.002	−0.001	0.003	0.005	0.003	0.009^*^	0.003	0.002	0.003
**FI** × **EA**	0.019^**^	0.004	0.019^**^	0.004	0.042^**^	0.004	0.000	0.004	0.005	0.004	0.004	0.004	0.015^*^	0.004	0.010^*^	0.004
**FI** × **Sex**	0.100^**^	0.015	0.127^**^	0.015	0.098^**^	0.015	0.012	0.014	0.012	0.015	0.185^**^	0.016	0.200^**^	0.016	0.162^**^	0.016

* *p < 0.01*,

** *p < 0.0001*.

*Using Bonferroni correction, the results that are significant on the 0.0001 level will be significant on the 0.005 level and the results that are significant on the 0.01 level will not be significant: coding of sex is 1 for males, 0 for females. Focal effects are in bold*.

It is clear that although the signs of all the significant interactions were in the hypothesized directions, there were quite a few interactions that were not significant. Although all the 14 significant interactions were consistent with our hypothesis, the remaining 10 interactions were not significant, neither supporting nor opposing the investment as interaction hypothesis.

The non-significant interactions are also of interest. Six of them are associated with verbal abilities, which is consistent with the idea that the investment of fluid intelligence is less important in the development of verbal abilities than the development of mathematical and science abilities. Three of the non-significant interactions involve techn**i**cal abilities as measures of crystalized intelligence and parents' education and educational aspirations as investment traits. Since these two traits are associated with socioeconomic background, these non-significant interactions, together with the significant interactions involving these two traits when the crystalized abilities are stochastic abilities, suggest that socioeconomic factors are associated primarily with a motivation for scholastic, rather than technical, achievement.

In addition to our focal interaction effect, an interesting pattern that emerges from our results is related to the quadratic effects of fluid intelligence, which was significantly positive for the two mathematical abilities and significantly negative for the other abilities. This pattern suggests an increasing marginal effect of fluid intelligence on mathematical abilities, but a diminishing marginal effect on the other abilities.

**Figure 2:**
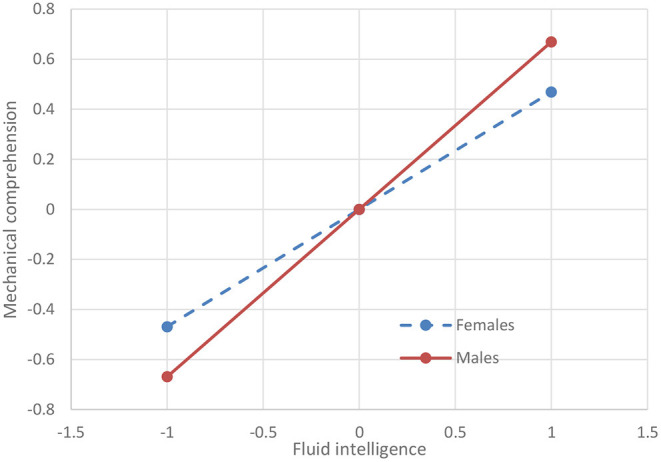
The effect of fluid intelligence on mechanical comprehension for males and females. Both fluid intelligence and mechanical comprehension are expressed in *Z* scores.

Finally, we note that the implications of the significant interactions are not negligible. This is particularly the case for the interactions between fluid intelligence and sex, where a standard deviation increase in fluid intelligence is associated with additional increases of up to 0.200 standard deviations in test scores for males vs. females. These additional increases for males are smaller for the scholastic tests (0.100, 0.127, and 0.098 standard deviations for arithmetic reasoning, general science, and math knowledge, respectively), and larger for the technical tests (0.185, 0.200, and 0.162 standard deviations for auto/shop information, mechanical comprehension, and electronic information, respectively). As an example, Figure 2 shows the effect of fluid intelligence on mechanical comprehension separately for males and females.

The implications of the significant interactions involving parents' education and educational aspirations are smaller. A one standard deviation increase in in fluid intelligence is associated with an additional increase of up to 0.084 standard deviation in test scores for a participant with high educational expectations (1std above the mean) vs. participants with low educational expectations (1std below the mean). For parents' education the increase is up to 0.026 standard deviations. It is also evident from Table 1 that the investment effect of educational aspirations is higher than the investment effect of parents' education, which is consistent with the idea the educational expectations mediate the effect of parents' education on crystalized intelligence.

## General Discussion

In the 1941 APA conference, Reymond Cattell (simultaneously with Donald Hebb, see Brown, 2016) presented a summary of his theory of intelligence suggesting that intelligence is made of two separate general factors, fluid intelligence, that represents thinking and reasoning processes associated primarily with tasks that require adaptation to new circumstances and crystalized intelligence that represents accessible knowledge and the ability to acquire additional knowledge using familiar learning strategies. Cattell suggested that babies are born with fluid ability which they use in their encounters with the world, thus forming their crystalized intelligence. In a subsequent work Kvist and Gustafsson (2008) suggested that this process depends on socio-economic factors. Children from high socio-economic background have more learning opportunities and therefore have better chances to rely on their fluid intelligence in the development of crystalized intelligence.

Building on Cattell's work, and on the work of earlier scholars who suggested that non-cognitive traits are involved in the development of intelligence (Thorndike et al., 1926; Wechsler, 1939; Vernon, 2014/1969). Ackerman and his co-authors (Ackerman, 1996, 2007; Chamorro-Premuzic et al., 2006; Von Stumm and Ackerman, 2013; von Stumm and Ackerman, 2014) suggested that investment traits, which they defined as “stable individual differences in the tendency to seek out, engage in, enjoy, and continuously pursue opportunities for effortful cognitive activity” (von Stumm et al., 2011, p. 225) underlie the potency by which fluid intelligence is invested in the formation of crystalized intelligence. Nevertheless, although this conceptualization of Cattell's investment theory has been the subject of many studies in the literature, most of them were based on a model in which the effects of fluid intelligence and investment characteristics on crystalized intelligence are additive (e.g., Von Stumm and Ackerman, 2013 for a review). Although there were a few articles that examined interactive effects between these two variables, their results were contradictory. Some found a facilitating relationship between the two, others found a compensatory relationship, while others did not found a significant interactive relationship (see Table 1).

Since an interaction model (Figure 1A) is a most natural interpretation of investment, it is interesting to ask why there were no more studies that examined Cattell's theory of investment from an interactionist approach. In our view, one reason is the low power in detecting interaction (Aguinis and Stone-Romero, 1997; Shieh, 2009). This is an especially severe problem when multi-collinearity between the components of the interaction is high (McClelland and Judd, 1993), which is usually the case with regard to the relationships between fluid intelligence and investment traits. The problem of biased, and even opposite sign coefficients when quadratic terms are not controlled for (Ganzach, 1997) may exacerbate the issue.

To solve this problem, we suggest relying on large representative databases that include information about fluid and crystalized intelligence, and using antecedents of interest that were not considered so far as moderators for the effect of fluid intelligence on crystalized intelligence. This suggestion is supported by the current results, but it is also supported by the fact that the few previous successful demonstrations of interactive relationships associated with fluid and crystalized intelligence were documented by analyzing large database, estimating the interaction between fluid intelligence and interest (Lechner et al., 2019), or demographic proxies of interest (Tucker-Drob and Briley, 2012). It is also supported by later development in Cattell's theory advanced by Kvist and Gustafsson (2008) who, relying on a factor analysis methodology (rather than on moderated multiple regression), suggested that investment depends on socio-economic factors, arguing that children from high socio-economic background have more learning opportunities and therefore better chances to utilize their fluid intelligence in the development of crystalized intelligence. Thus, future research on investment would benefit most from using large datasets and controlling for quadratic terms and relying on antecedents of interest, which, unlike personality/motivational investment trait, are usually available in the large datasets that are appropriate to studying the relationship between fluid and crystalized intelligence. In addition, replications of the current study in using other measures of fluid and crystalized intelligence, more recent data, longitudinal designs and additional populations will help in establishing the extent to which the current results are generalizable. On the one hand, crystalized intelligence had become more and more important for success in today's economy, which may have led to increased motivation to investing fluid intelligence in the formation of crystalized intelligence. On the other hand, the interaction effects (as well as the main effects) involving sex may have become weaker given the efforts made in recent years to raise more interest into STEM fields among females. Thus, the effects of various antecedent of interest on the effect of fluid intelligence on the formation of crystalized intelligence may had changed and are clearly an interesting topic for future research.

## Data Availability Statement

The original contributions presented in the study are included in the article/Supplementary Material, further inquiries can be directed to the corresponding author/s.

## Ethics Statement

The studies involving human participants were reviewed and approved by Ariel. The patients/participants provided their written informed consent to participate in this study.

## Author Contributions

YG initiated the study and analyzed the data, conceptualized the design, and wrote the paper.

## Funding

This work was supported by University grant.

## Conflict of Interest

The author declares that the research was conducted in the absence of any commercial or financial relationships that could be construed as a potential conflict of interest.

## Publisher's Note

All claims expressed in this article are solely those of the authors and do not necessarily represent those of their affiliated organizations, or those of the publisher, the editors and the reviewers. Any product that may be evaluated in this article, or claim that may be made by its manufacturer, is not guaranteed or endorsed by the publisher.

## Footnotes

^1^The OFCI model has also other propositions about the relationships between openness to experience, fluid intelligence and crystalized intelligence which are not relevant to the current discussion.

^2^Interestingly enough, in their study, Lechner et al. (2019) did not find an interactive effect for the other investment trait that they studied, openness to experience (although they found a linear effect for this trait), which further emphasize the need for large samples to detect the interactive effects involving fluid intelligence.

^3^Studies that examined the Matthew effect (i.e., “the rich get richer,” see Merton, 1968) are also relevant here since they often deal with facilitating effects involving socioeconomic status (see for example Judge and Hurst, 2008). In particular, based on a large sample (*n* = 81,000) Damian et al. (2015) showed a facilitating relationship between intelligence and parental socioeconomic status in determining status attainment. Although this study is not about the relationship between fluid and crystalized intelligence, which is the focus of the current paper, it is relevant to our work since it also has to do with interactive relationships involving socioeconomic status and intelligence in predicting achievement.

^4^We also conducted robustness checks by estimating regressions for each of two the components of our measure of fluid intelligence. The results of these regressions are presented in the Appendix in Supplementary Material, and it is evident that they are rather similar to the results of the composite measure. There is, however, an interesting difference between the two sets of regression. For coding speed, but not for numerical operations, we observe negative interactions between fluid intelligence and sex in determining verbal abilities (word knowledge and paragraph comprehension), suggesting that females, more than males, invest their fluid intelligence in the acquisition of verbal abilities, which is consistent with findings indicating that females are more interested in verbal skills (Su et al., 2009). Thus it seems that for analyzing the investment aspects of sex, coding speed may be a better indicator of fluid intelligence than numerical operations.
