# Supplementary material for: Antecedents of Interest and the Investment of Fluid Intelligence in the Formation of Crystalized Intelligence
Source: Front Psychol. 2021 Oct 4;12:679504. doi: 10.3389/fpsyg.2021.679504 (PMC8521038; doi:10.3389/fpsyg.2021.679504)
Supplement: Supplementary file 1 [file Table_1.docx]

**Table A1: Regressions of crystalized abilities where fluid intelligence is measured by numerical operations**

|  | **Math/science abilities** | | | | | | **Verbal abilities** | | | | **Technical abilities** | | | | | |
| --- | --- | --- | --- | --- | --- | --- | --- | --- | --- | --- | --- | --- | --- | --- | --- | --- |
|  | **General**  **science** | | **Arithmetic**  **reasoning** | | **Math**  **knowledge** | | **Word**  **knowledge** | | **Paragraph**  **comprehension** | | **Auto/shop**  **information** | | **Mechanical**  **comprehension** | | **Electronic**  **information** | |
|  | **b** | **sderr** | **b** | **sderr** | **b** | **sderr** | **b** | **sderr** | **b** | **sderr** | **b** | **sderr** | **b** | **sderr** | **b** | **sderr** |
| Intercept | -2.228 | 0.637 | -0.498 | 0.618 | -1.557 | 0.610 | -2.701 | 0.591 | -3.304 | 0.622 | -4.248 | 0.646 | -2.327 | 0.679 | -2.829 | 0.657 |
| Fluid intelligence (Fl) | 0.184 | 0.061 | 0.287 | 0.059 | -0.221 | 0.054 | 0.385 | 0.053 | 0.369 | 0.056 | 0.115 | 0.058 | 0.006 | 0.061 | 0.137 | 0.059 |
| Parents’ education (PE) | 0.034 | 0.038 | -0.005 | 0.037 | 0.010 | 0.036 | 0.024 | 0.035 | 0.057 | 0.037 | 0.141 | 0.038 | 0.089 | 0.040 | 0.011 | 0.039 |
| Education aspiration (EA) | -0.348 | 0.014 | -0.320 | 0.013 | 0.152 | 0.013 | 0.072 | 0.013 | -0.082 | 0.013 | 0.949 | 0.014 | 0.704 | 0.015 | 0.664 | 0.014 |
| Sex | 0.082 | 0.060 | -0.021 | 0.058 | 0.030 | 0.057 | 0.077 | 0.055 | 0.156 | 0.058 | 0.145 | 0.061 | 0.022 | 0.064 | 0.076 | 0.062 |
| Age | 0.058 | 0.009 | 0.014 | 0.009 | 0.001 | 0.009 | 0.055 | 0.009 | 0.056 | 0.009 | 0.075 | 0.009 | 0.038 | 0.010 | 0.063 | 0.010 |
| FI^2^ | -0.076 | 0.007 | 0.039 | 0.007 | 0.050 | 0.007 | -0.104 | 0.007 | -0.083 | 0.007 | -0.072 | 0.007 | -0.038 | 0.008 | -0.049 | 0.007 |
| PE^2^ | 0.001 | 0.001 | 0.002 | 0.001 | 0.003 | 0.001 | 0.001 | 0.001 | 0.000 | 0.001 | -0.005 | 0.001 | -0.003 | 0.001 | 0.001 | 0.001 |
| EA^2^ | -0.001 | 0.002 | 0.001 | 0.001 | -0.001 | 0.001 | 0.000 | 0.001 | -0.003 | 0.001 | -0.001 | 0.002 | 0.001 | 0.002 | 0.000 | 0.002 |
| Age^2^ | 0.001 | 0.000 | 0.002 | 0.000 | 0.003 | 0.000 | 0.001 | 0.000 | 0.000 | 0.000 | -0.001 | 0.000 | 0.001 | 0.000 | 0.000 | 0.000 |
| **FI X PE** | **0.008*** | 0.003 | **0.015**** | 0.002 | 0.013****** | 0.002 | 0.005 | 0.002 | 0.002 | 0.002 | 0.008***** | 0.003 | **0.010**** | 0.003 | 0.004 | 0.003 |
| **FI X EA** | **0.017**** | 0.004 | **0.018**** | 0.004 | 0.040****** | 0.004 | -0.001 | 0.004 | 0.005 | 0.004 | 0.002 | 0.004 | **0.012*** | 0.004 | 0.008 | 0.004 |
| **FI X Sex** | **0.083**** | 0.014 | **0.089**** | 0.014 | 0.057****** | 0.013 | 0.010 | 0.013 | 0.011 | 0.014 | 0.174****** | 0.014 | **0.169**** | 0.015 | **0.142**** | 0.014 |

*p<.01, **p<.0001

Using Bonferioni correction, the results that are significant on the .0001 level will be significant on the .005 level and the results that are significant on the .01 level will not be significant

**Table A2: Regressions of crystalized abilities where fluid intelligence is measured by coding speed**

|  | **Math/science abilities** | | | | | | **Verbal abilities** | | | | **Technical abilities** | | | | | |
| --- | --- | --- | --- | --- | --- | --- | --- | --- | --- | --- | --- | --- | --- | --- | --- | --- |
|  | **General**  **science** | | **Arithmetic**  **reasoning** | | **Math**  **knowledge** | | **Word**  **knowledge** | | **Paragraph**  **comprehension** | | **Auto/shop**  **information** | | **Mechanical**  **comprehension** | | **Electronic**  **information** | |
|  | **b** | **sderr** | **b** | **sderr** | **b** | **sderr** | **b** | **sderr** | **b** | **sderr** | **b** | **sderr** | **b** | **sderr** | **b** | **sderr** |
| Intercept | -2.479 | 0.648 | -1.089 | 0.652 | -1.286 | 0.641 | -2.338 | 0.611 | -2.751 | 0.636 | -3.928 | 0.648 | -2.009 | 0.680 | -2.564 | 0.663 |
| Fluid intelligence (Fl) | 0.332 | 0.055 | 0.216 | 0.056 | -0.073 | 0.055 | 0.667 | 0.052 | 0.676 | 0.054 | 0.386 | 0.055 | 0.220 | 0.058 | 0.349 | 0.057 |
| Parents’ education (PE) | -0.038 | 0.038 | -0.076 | 0.038 | -0.092 | 0.038 | -0.029 | 0.036 | -0.022 | 0.037 | 0.092 | 0.038 | 0.030 | 0.040 | -0.042 | 0.039 |
| Education aspiration (EA) | 0.411 | 0.014 | 0.395 | 0.014 | 0.219 | 0.014 | 0.140 | 0.014 | -0.008 | 0.014 | 1.009 | 0.014 | 0.774 | 0.015 | 0.726 | 0.015 |
| Sex | 0.093 | 0.061 | 0.024 | 0.061 | 0.075 | 0.060 | 0.077 | 0.057 | 0.160 | 0.060 | 0.155 | 0.061 | 0.040 | 0.064 | 0.092 | 0.062 |
| Age | 0.034 | 0.009 | -0.006 | 0.009 | -0.018 | 0.009 | 0.035 | 0.009 | 0.034 | 0.009 | 0.054 | 0.009 | 0.019 | 0.010 | 0.045 | 0.009 |
| FI^2^ | -0.044 | 0.007 | 0.027 | 0.007 | 0.031 | 0.006 | -0.064 | 0.006 | -0.062 | 0.006 | -0.041 | 0.007 | -0.021 | 0.007 | -0.034 | 0.007 |
| PE^2^ | 0.004 | 0.001 | 0.005 | 0.001 | 0.007 | 0.001 | 0.004 | 0.001 | 0.003 | 0.001 | -0.003 | 0.001 | 0.000 | 0.001 | 0.003 | 0.001 |
| EA^2^ | -0.001 | 0.002 | 0.000 | 0.002 | -0.002 | 0.002 | 0.000 | 0.001 | -0.003 | 0.002 | -0.002 | 0.002 | 0.000 | 0.002 | 0.000 | 0.002 |
| Age^2^ | 0.002 | 0.000 | 0.003 | 0.000 | 0.004 | 0.000 | 0.002 | 0.000 | 0.001 | 0.000 | 0.000 | 0.000 | 0.002 | 0.000 | 0.001 | 0.000 |
| **FI X PE** | -0.004 | 0.002 | 0.006 | 0.003 | **0.007**** | 0.002 | **-0.007*** | 0.002 | **-0.009*** | 0.002 | -0.004 | 0.002 | 0.002 | 0.003 | -0.005 | 0.003 |
| **FI X EA** | 0.003 | 0.004 | **0.009*** | 0.004 | **0.026**** | 0.004 | **-0.013**** | 0.004 | **-0.010*** | 0.004 | -0.008 | 0.004 | 0.003 | 0.004 | -0.001 | 0.004 |
| **FI X Sex** | **0.097**** | 0.015 | **0.122**** | 0.015 | **0.109**** | 0.015 | 0.021 | 0.014 | 0.026 | 0.015 | **0.191**** | 0.015 | **0.197**** | 0.016 | **0.152**** | 0.015 |

*p<.01, **p<.0001

Using Bonferioni correction, the results that are significant on the .0001 level will be significant on the .005 level and the results that are significant on the .01 level will not be significant
